# Association of Timing of School Desegregation in the United States With Late-Life Cognition in the Study of Healthy Aging in African Americans (STAR) Cohort

**DOI:** 10.1001/jamanetworkopen.2021.29052

**Published:** 2021-10-20

**Authors:** Rachel L. Peterson, Kristen M. George, Lisa L. Barnes, Paola Gilsanz, Elizabeth Rose Mayeda, M. Maria Glymour, Dan M. Mungas, Rachel A. Whitmer

**Affiliations:** 1 Department of Public Health Sciences, University of California, Davis; 2 Department of Neurological Sciences, Rush University, Chicago, Illinois; 3 Kaiser Permanente Northern California Division of Research, Oakland; 4 Fielding School of Public Health, University of California, Los Angeles; 5 Department of Epidemiology and Biostatistics, University of California, San Francisco; 6 Department of Neurology, University of California, Davis

## Abstract

**Importance:**

Prior research suggests schooling differences for Black individuals in the US are associated with worse cognitive aging. It is unknown whether age when experiencing school desegregation is associated with differences in late-life cognition in this population.

**Objective:**

To examine patterns of association between age of school desegregation in grades 1 to 12 and late-life cognition.

**Design, Setting, and Participants:**

This cohort study analyzed baseline data from the Study of Healthy Aging in African Americans (STAR) cohort collected from 2018 through 2019 in Northern California, primarily in the cities of Richmond and Oakland. Participants were 699 self-identified Black individuals aged 50 years or older who were community-dwelling, long-term members of Kaiser Permanente Northern California and dementia free at baseline.

**Exposures:**

Participants reported whether they attended a segregated school in grades 1, 6, 9, and 12 and were placed in 1 of 6 transition categories: (1) always attended integrated schools; (2) integrated between grades 1 through 5; (3) integrated between grades 6 through 8; (4) integrated between grades 9 through 12; (5) ever moved from integrated to segregated school; (6) never attended integrated schools.

**Main Outcomes and Measures:**

Executive function, semantic memory, and verbal episodic memory ascertained via the Spanish and English Neuropsychological Assessment Battery and *z* standardized for analyses.

**Results:**

The mean (SD) age of the 699 participants was 68.5 (8.7) years, and 484 (69.2%) were female. Most participants transitioned from segregated to integrated schools owing to historical timing and cohort geography. Compared with 111 participants who never attended integrated schools (reference group), executive function was better among 50 participants who transitioned to integrated schools between grades 1 and 5 (β = 0.35; 95% CI, 0.08-0.61; *P* = .01). Semantic memory was better among 435 participants who only attended integrated schools (β = 0.34; 95% CI, 0.14-0.54; *P* = .001) or among 50 participants who transitioned to integrated schools between grades 1 and 5 (β = 0.43; 95% CI, 0.15-0.72; *P* = .003). However, no significant differences were found by group for verbal episodic memory function (eg, for 50 participants who transitioned to integrated schools between grades 1 and 5: β = 0.07; 95% CI, −0.22 to 0.35; *P* = .66). No significant differences were found when testing for potential interactions by sex, Southern birth, or baseline age.

**Conclusions and Relevance:**

The STAR cohort data indicated that executive function and semantic memory were higher among Black individuals with some integrated school experience. These results suggest that racially segregated schooling experiences, including de facto segregation present today, may be associated with worse late-life cognition.

## Key Points

**Question:**

Is the timing of change from attendance at a de facto or de jure racially segregated school to an integrated school associated with late-life cognition among Black individuals in the US?

**Findings:**

In this cohort study of 699 self-identified Black participants, those who only transitioned to an integrated school between first and sixth grades had statistically significantly better executive function and semantic memory than those who only attended segregated schools. No difference by group was found for verbal episodic memory.

**Meaning:**

Segregated school attendance may be associated with worse late-life cognition among older Black individuals in the US, a pattern that may also be present among younger generations who attend de facto segregated schools.

## Teaser

This cohort study assesses whether school desegregation experience or its timing is associated with cognitive function in middle or late life among Black individuals residing in Northern California.

## Introduction

Studies in the literature have found marked racial and ethnic disparities in cognitive aging and dementia.^1,2^ Many factors may contribute to these observed disparities, such as individual socioeconomic differences, as well as structural and institutional factors shaped by social norms and policy. Educational attainment and quality have consistently been observed to partially explain racial differences in late-life cognitive performance and dementia risk.^3,4,5,6,7,8,9^ Educational experiences in the US have historically been vastly different for Black students and White students. Schools in numerous states across the country were legally segregated (ie, de jure segregation) until the 1954 Supreme Court ruling in *Brown vs Board of Education* declared that “separate but equal” school segregation was unconstitutional.^10^ Nonetheless, many schools resisted integration until the 1964 Civil Rights Act.^11^ Persistent residential and economic segregation resulting from institutionalized racism continues to reinforce de facto racial segregation of many public schools across the US today, making any potential cognitive outcomes of segregated school experiences pertinent for current and future generations.^12,13,14^

Only a handful of studies have examined US school segregation experiences in the context of late-life cognition. One study found that attending integrated schools was associated with better late-life cognition,^15^ whereas 2 studies have found that older Black individuals who attended integrated schools had worse cognitive outcomes than older Black individuals who attended only segregated schools.^16,17^ These discrepancies may be due to differences in the cohorts, such as age or where the participants attended school (ie, the South vs Northeast or Midwest). The experiences of older participants who attended a de jure segregated school in the South likely differ from those of younger participants who attended a de facto segregated school in the Midwest in ways that may be associated with late-life cognition.

Education and cognition are inextricably linked; the purpose of education is to engender cognitive skills at any age. However, the association between educational attainment and later-life cognitive outcomes may also be due to the association of education with many modifiable risk and protective factors for cognitive aging. One hypothesis is that education shapes cognition through its role in producing denser neurocognitive networks that allow for greater adaptability and resilience in the presence of neuropathologic conditions, such as Alzheimer disease.^18,19^ Differences in school quality and years of educational attainment associated with segregated schools may help to explain some disparities in health generally^20^ and cognitive aging specifically.^4,21,22^ For example, Ashenfelter et al^11^ found that among Southern-born Black students, those who finished schooling a few years prior to integration had poorer economic and employment outcomes than their counterparts completing school a few years after integration, which was partially associated with increased years of schooling among the latter group. However, the quality gap between Black schools and White schools had been diminishing in the decades prior to *Brown vs Board of Education* and the Civil Rights Act as a result of lawsuits and private philanthropy.^11,23^

The specific experience of school integration may also be negatively associated with late-life cognition additionally or alternatively through psychosocial stress because many Black students in newly integrated schools often faced discrimination and violence. Furthermore, segregated schools attended by Black students were likely staffed by Black teachers, whereas integrated schools were predominantly staffed by White teachers. Students leaving a segregated school may have thus lost important role models or social connections with race-concordant mentors when removed to an integrated school. Neurodevelopment in adolescence accelerates in many brain regions associated with poor cognitive functioning in late life, creating a sensitive period whereby exposure to adolescent stress may permanently negatively alter adult cognitive abilities and increase dementia risk.^24,25,26^ School quality and psychosocial stress pathways may not be mutually exclusive, and they may be most relevant at different age periods of school attendance. The capacity to learn may be enhanced in school settings free of hostility and enriched by a stronger sense of community, even if fewer monetary resources were available in segregated schools. Depending on the life course timing and historical timing of the school integration experience, school quality, psychosocial stress, or both may be pertinent pathways associated with late-life cognition.

The present study adds to the literature by examining how school desegregation experiences, and the timing of those experiences in the life course, are associated with cognitive functioning measured in middle and late life in a cohort of Black individuals residing in California. By examining the diverse educational experiences in a Black-only sample, we can further disentangle how educational experiences may be associated with cognitive outcomes in this population. We hypothesize that Black individuals who experienced school integration during their K-12 school academic years will have worse cognitive outcomes associated with the stress from this transition and the exposure to greater hostility and violence in the integrated school environment compared with Black individuals who never attended an integrated school.

## Methods

### Sample

The Study of Healthy Aging in African Americans (STAR) cohort includes community-dwelling older adults residing in Northern California, primarily the cities of Oakland and Richmond. The Study of Healthy Aging in African Americans aims to evaluate how life course vascular and sociocultural factors are associated with the trajectory of cognitive aging and the burden of cognitive impairment among Black individuals in the US. Individuals eligible for STAR were long-term members of Kaiser Permanente Northern California (KPNC)—an integrated health care delivery system—who self-identified as Black, were aged 50 years or older on January 1, 2018, and had previously participated in KPNC multiphasic health checkup examinations between 1964 and 1985. Stratified random sampling by age and educational attainment was used with the goal of recruiting approximately equal proportions of participants aged 50 to 64 years and participants aged 65 years or older. Exclusion criteria included electronic medical record diagnosis of dementia or other neurodegenerative disease (eg, frontotemporal dementia, Lewy body disease, Pick disease, Parkinson disease with dementia, and Huntington disease) and presence of health conditions that would impede participation in study interviews (defined as hospice activity in the last 12 months, history of severe chronic obstructive pulmonary disease in the last 6 months, congestive heart failure hospitalizations in the past 6 months, and history of kidney failure or dialysis in the last 12 months). Baseline interviews occurred at participants’ homes or at KPNC clinics between November 2017 and March 2020. Baseline data were collected from 2018 through 2019. Reporting followed the Strengthening the Reporting of Observational Studies in Epidemiology (STROBE) reporting guideline for observational studies. The study was approved by the KPNC and University of California, Davis institutional review boards. All enrolled participants provided written informed consent that was obtained in a manner consistent with the Common Rule requirements. The STAR participants received a $50 check for participation after the completion of a third study visit. At each study visit, participants also received a promotional item (ie, chip clip, pen, or small golf towel) with the study logo.

### Measures

#### Cognition

Cognitive function was assessed using the Spanish and English Neuropsychological Assessment Scales (SENAS), a battery of cognitive tests that has undergone extensive development using item response theory methods for valid comparisons of cognition and cognitive change across racial and ethnic and linguistically diverse groups. Three cognitive domains (executive function, verbal episodic memory, and semantic memory) were derived from the SENAS, and each domain was *z* standardized using the mean (SD) values from the full baseline sample. Details of the administration procedures, development, and psychometric characteristics have been described in detail elsewhere.^27,28^

#### Timing of School Integration Experience

Participants were asked if they attended school in 1st, 6th, 9th, and 12th grades and whether that school was segregated. We used the change in participant responses from one grade to the next to indicate the life-course timing of desegregation. Accordingly, participants were classified into 1 of 6 categories: (1) always attended integrated schools; (2) integrated between 1st and 5th grades; (3) integrated between 6th and 8th grades; (4) integrated between ninth 9th and 12th grades; (5) ever moved from integrated to segregated school; and (6) never attended integrated schools. Participants with more than 1 transition (eg, reported attending a segregated school in first grade, integrated in sixth grade, and segregated in ninth grade) were classified according to their first transition. Participants were excluded from analysis if they were missing information on school attendance or segregation for 3 or more grades.

#### Other Measures

Covariates were captured from STAR wave 1 and included participant age, sex (male vs female), educational attainment, and birth state. Educational attainment was reported as the last or highest level of school completed for credit and recoded as a continuous variable of estimated years of completed education (eg, bachelor’s degree = 16) for analyses. Birth state was coded into regions as South vs other using the US Census Bureau regional designations (eTable 1 in the Supplement).^29^

### Statistical Analysis

Participant demographic characteristics are reported overall and stratified by category of school integration experiences. Using linear regression, we examined the association between the timing of the school integration experience and late-life cognition (domain specific). Potential associated factors dichotomized by age at the 50th percentile (66.5 years), sex, and birth region (Southern vs other) were examined using interaction terms in separate models. For each cognitive domain, model 1 adjusted for age; model 2 adjusted for age, sex, and educational attainment; and model 3 adjusted for age, sex, educational level, and birth region dichotomized as Southern vs other.

We performed 3 sensitivity analyses. First, we examined potential bias from excluding participants who were missing school integration information by comparing demographic differences with our primary sample and including these participants in fully adjusted models. Our second sensitivity analysis additionally controlled for childhood hunger, childhood family finances, and parental educational level to examine whether other childhood exposures changed the main effects of segregated school attendance. Finally, we excluded participant educational attainment from the primary models. Educational attainment may be influenced by segregation, and adjusting for educational level may underestimate the association of attending segregated schools in our primary models. However, adjustment for educational attainment may also help isolate factors associated with psychosocial or school quality consequences. All hypothesis tests were 2-sided with statistical significance levels at *P* ≤ .05. Analyses were performed using Stata, version 14.2 (StataCorp).

## Results

From a cohort of 764 participants, we excluded 49 participants for missing information on school segregation at 3 or more grades, 13 participants for missing birth state, and 3 participants for missing level of completed education. Our analytic sample consisted of 699 participants with a mean (SD) age of 68.5 (8.7) years (range, 53-95 years), and 484 participants (69.2%) were female, whereas 215 (30.8%) were male (Table 1). Of 699 participants, 264 (37.8%) attended a de jure or de facto segregated school at some point between 1st and 12th grades. Participants who never attended integrated schools tended to be older (mean [SD] age, 75.0 [8.6] years), whereas participants who only attended integrated schools (mean [SD] age, 72.0 [9.3] years) or who transitioned from an integrated school to a segregated school (mean [SD] age, 65.8 [7.0] years) tended to be younger (*P* < .001). Participants had a mean (SD) of 14.5 (2.4) years of education, with no significant differences observed in educational attainment by school desegregation experience. Approximately one-third of participants (257 [36.8%]) were born in the South. Differences by birth region were observed in school segregation experiences (*P* < .001), with 86 of 111 participants (77.5%) born in the South reporting that they never attended an integrated school, and 363 of 435 participants (83.4%) born in the west, Midwest, or Northeast reporting that they only attended integrated schools. This pattern may be associated with secular trends in education and migration because 218 of 407 participants aged 65 years or older (53.6 %) were born in the South compared with 39 of 292 participants younger than 65 years (13.4%).

**Table 1.** Baseline Characteristics of STAR Analytic Sample, by School Segregation Experience

Characteristic	Mean (SD) values							*P* value, test of difference
	Overall (n = 699)	Attended only integrated schools (n = 435)	Integrated			Changed from integrated to segregated (n = 26)	Never attended integrated schools (n = 111)	
			Between 1st and 5th grades (n = 50)	Between 6th and 8th grades (n = 48)	Between 9th and 12th grades (n = 29)			
Age (range, 53-95), y	68.5 (8.7)	65.8 (7.1)	72.0 (9.3)	72.8 (9.2)	72.7 (10.2)	65.8 (7.0)	75.0 (8.6)	<.001^a^
Female sex, No. (%)	484 (69.2)	304 (69.9)	38 (76.0)	32 (66.7)	21 (72.4)	18 (69.2)	71 (64.0)	.72^b^
Educational level (range: 9-20 y), y	14.5 (2.4)	14.6 (2.3)	14.3 (2.4)	13.9 (2.3)	14.5 (2.4)	14.7 (2.4)	14.6 (2.4)	.50^a^
Birth region, No. (%)								<.001^b^
South	257 (36.8)	72 (16.6)	32 (64.0)	34 (70.8)	22 (75.9)	11 (42.3)	86 (77.5)	
West, Midwest, or Northeast	442 (63.2)	363 (83.5)	18 (36.0)	14 (29.2)	7 (24.1)	15 (57.7)	25 (22.5)	
Late-life cognition								
Executive function	0.04 (0.97)	0.22 (0.86)	0.06 (1.08)	−0.43 (1.11)	−0.25 (1.02)	0.13 (0.80)	−0.42 (1.02)	<.001^a^
Semantic memory	0.05 (0.97)	0.25 (0.88)	0.01 (1.00)	−0.48 (0.95)	−0.24 (0.99)	0.35 (0.87)	−0.45 (1.03)	<.001^a^
Verbal memory	0.04 (0.98)	0.17 (0.92)	−0.07(1.05)	−0.28 (1.02)	0.10 (0.88)	0.22 (0.74)	−0.30 (1.16)	<.001^a^

Abbreviation: STAR, Study of Healthy Aging in African Americans.

^a^ Using the χ^2^ test of significance.

^b^ One-way analysis of variance.

Compared with 111 participants who never attended an integrated school, 435 participants who only attended integrated schools had significantly better semantic memory (β = 0.34; 95% CI, 0.14-0.54; *P* = .001), albeit no statistically significant differences were observed for executive function (β = 0.15; 95% CI, −0.04 to 0.34; *P* = .12) or verbal episodic memory (β = 0.00; 95% CI, −0.21 to 0.21; *P* = .99) (Table 2). Among 50 participants who transitioned from segregated to integrated schools between first and fifth grades, we observed higher semantic memory (β = 0.43; 95% CI, 0.15-0.72; *P* = .003) and executive function (β = 0.35; 95% CI, 0.08-0.61; *P* = .01) but not verbal episodic memory (β = 0.07; 95% CI, −0.22 to 0.35; *P* = .66). There was no significant difference in any cognitive domain between participants who transitioned from segregated to integrated schools between 6th and 8th grades (n = 48) or between 9th and 12th grades (n = 29) and participants who never attended integrated schools. We observed better semantic memory (β = 0.55; 95% CI, 0.17-0.92; *P* = .004) among 26 participants who transitioned from integrated to segregated schools at some point in their K-12 school career. We observed no significant interactions of school segregation or integration experience by sex, age, or Southern birth. Our sensitivity analyses further confirmed these findings (eTables 2, 3, 4, and 5 in the Supplement).

**Table 2.** Linear Regression Model Coefficients for the Association Between Segregated/Integrated School Attendance Experience and *z*-Standardized Late-Life Cognitive Domains in STAR

Model	Executive function		Semantic memory		Verbal episodic memory	
	β (95% CI)	*P* value	β (95% CI)	*P* value	β (95% CI)	*P* value
**Adjusted for age**						
Attended integrated schools						
Never	1 [Reference]		1 [Reference]		1 [Reference]	
Only	0.20 (0.004 to 0.39)	.05	0.46 (0.25 to 0.66)	<.001	0.05 (−0.15 to 0.26)	.62
Integrated between						
1st and 5th grades	0.34 (0.05 to 0.62)	.02	0.38 (0.07 to 0.68)	.02	0.11 (−0.20 to 0.41)	.50
6th and 8th grades	−0.18 (−0.47 to 0.11)	.21	−0.09 (−0.40 to 0.22)	.58	−0.08 (−0.38 to 0.23)	.63
9th and 12th grades	0.05 (−0.29 to 0.40)	.76	0.15 (−0.22 to 0.52)	.42	0.30 (−0.07 to 0.67)	.11
From integrated to segregated schools	0.10 (−0.27 to 0.47)	.60	0.56 (0.16 to 0.96)	.01	0.11 (−0.29 to 0.50)	.60
**Adjusted for age, sex, and educational level**						
Attended integrated schools						
Never	1 [Reference]		1 [Reference]		1 [Reference]	
Only	0.19 (0.01 to 0.37)	.04	0.47 (0.28 to 0.66)	<.001	0.04 (−0.16 to 0.23)	.71
Integrated between						
1st and 5th grades	0.36 (0.09 to 0.62)	.01	0.46 (0.17 to 0.74)	.002	0.07 (−0.22 to 0.36)	.63
6th and 8th grades	−0.10 (−0.37 to 0.17)	.47	−0.01 (−0.30 to 0.28)	.96	−0.04 (−0.33 to 0.25)	.80
9th and 12th grades	0.06 (−0.26 to 0.38)	.71	0.20 (−0.14 to 0.55)	.25	0.27 (−0.08 to 0.62)	.13
From integrated to segregated schools	0.09 (−0.25 to 0.44)	.60	0.59 (0.21 to 0.97)	.002	0.09 (−0.28 to 0.46)	.63
**Adjusted for age, sex, educational level, and Southern birth**						
Attended integrated schools						
Never	1 [Reference]		1 [Reference]		1 [Reference]	
Only	0.15 (−0.04 to 0.34)	.12	0.34 (0.14 to 0.54)	.001	−0.001 (−0.21 to 0.21)	.99
Integrated between						
1st and 5th grades	0.35 (0.08 to 0.61)	.01	0.43 (0.15 to 0.72)	.003	0.07 (−0.22 to 0.35)	.66
6th and 8th grades	−0.10 (−0.37 to 0.17)	.46	−0.01 (−0.30 to 0.28)	.94	−0.04 (−0.33 to 0.25)	.79
9th and 12th grades	0.06 (−0.26 to 0.39)	.70	0.21 (−0.13 to 0.56)	.23	0.27 (−0.08 to 0.62)	.12
From integrated to segregated schools	0.07 (−0.27 to 0.42)	.67	0.55 (0.17 to 0.92)	.004	0.08 (−0.30 to 0.45)	.69

Abbreviation: STAR, Study of Healthy Aging in African Americans.

## Discussion

In our cohort of self-identified Black individualss aged 50 years or older who were long-term residents of Northern California, 35% attended a de jure or de facto segregated school at some point between 1st and 12th grades. Contrary to our hypothesis, we found that, compared with participants who never attended integrated schools, participants who transitioned to an integrated school between first and sixth grades had the highest executive function scores and significantly better semantic memory. We also observed better semantic memory among participants who only attended integrated schools compared with participants who only attended segregated schools. No significant differences in verbal episodic memory were found in our analyses. This may be because semantic memory and executive function may be more greatly associated with education and early life experiences, whereas verbal episodic memory is not. Although we detected better semantic memory among participants who transitioned from integrated to segregated schools, this finding should be interpreted cautiously owing to the small sample size.

Our findings add to the few studies that have examined the role of school segregation experiences for late-life cognitive outcomes among Black individuals and is the first study, to our knowledge, to evaluate life-course timing of school integration. Some of our findings are consistent with those of Aiken-Morgan et al,^15^ in that participants who only attended integrated schools had higher cognition than those who only attended segregated schools. The authors theorized that better school quality for integrated schools compared with segregated schools led to better cognitive function. However, if school quality were the only associated factor in our study, we would have expected to see a dose-response relationship, with integration experiences occurring earlier in the life course associated with better cognition. Although this association was observed among participants who integrated between 1st and 5th grades, we observed a pattern of higher point estimates (albeit nonsignificant) among those who transitioned to an integrated school between 8th and 12th grades than those who transitioned between 6th and 8th grades.

However, Lamar et al^17^ observed better late-life cognition among participants attending segregated compared with integrated schools. The authors theorized that their findings may be explained by the psychosocial stress experienced by individuals and communities during school integration processes. Our findings are also consistent with this theory because we observed negative point estimates and no statistical difference in cognition between participants who integrated between sixth and eighth grades and those who never attended integrated schools. The period of adolescence that typically coincides with sixth to eighth grades is considered a sensitive period in neurocognitive development that may be more greatly influenced by stress exposures.^24,25,26^ Educational quality and psychosocial stress pathways may be linked. Specifically, if integrated school environments were hostile, learning was likely stifled regardless of the level of school resources.

### Strengths and Limitations

Our study has several strengths. First, structural racism is recognized as key to the explanation of racial inequalities in health, although few studies have explicitly examined the potential associations with cognitive aging. Our study is among the first to examine how variations in experience of one form of structural racism—school segregation—is associated with late-life outcomes. The STAR cohort provides a unique opportunity for this investigation because participants are long-time residents of Northern California and encompass a wide age range of middle and late life. Our sample is particularly compelling because, as long-term members of Kaiser Permanente, mechanisms such as inadequate health insurance are unlikely to explain the associations. Our robust measure of cognitive domains via the SENAS allows for highly sensitive differentiation of cognition among racially and educationally diverse and cognitively intact individuals. In addition, this study had measures of both de facto and de jure segregated school experiences at multiple grades, which makes our findings pertinent for both current and future generations.

This study had limitations. First, only 1 wave of cognitive function assessments, gathered after the age of 50 years, is included. Cognitive data from early life and longitudinally in late life could help disentangle whether observed differences by school desegregation experience are the result of stable differences in cognition cemented in early adulthood or whether early life experiences shape trajectories of cognitive decline. Future waves of data collection will permit further examination of late-life cognitive trajectories. Second, our cohort—like most observational studies of older adults—is subject to selection and survival biases. Specifically, exclusion of individuals with a known dementia diagnosis may have contributed to selection bias, while there may have been differential survival among groups with different integration experiences that bias our findings. Our estimates also should be interpreted recognizing potential confounders and uncertain mechanisms. Specifically, we could not adjust for school details, including state of each attended school or other aspects of the school environment (eg, racial composition of teachers). Nonetheless, we were able to adjust for birth region (Southern vs non-Southern), which we expect reduces residual confounding due to discriminatory experiences outside of the school environment or other early life contextual factors that increase dementia risk.^30^ We further acknowledge the potential risk of misclassification of participants by school integration experience due to recall bias. However, unlike some early life exposures, school integration experience is likely relatively robust to recall because it would have been easily assessed by the child and, given the national controversy about school desegregation and the degree of racial stratification when these individuals were children, would likely have been noted. In addition, our exclusion of those with a dementia diagnosis provides some confidence that people could accurately recall this consequential and often traumatic early life experience. Finally, we acknowledge that variations in findings across studies, including ours, may be associated with differences in years of schooling, geographic origins, migration patterns, and other cohort factors. We were unable to simultaneously explore cohort effects and life-course timing of school integration experiences owing to statistical power limitations.

## Conclusions

Our findings help to explain how a historical process experienced by Black individuals in early life may contribute to poor cognitive aging outcomes. Our findings may be pertinent for understanding—and intervening on—the cognitive outcomes of future generations. Persistent residential and economic segregation continues to contribute to de facto school segregation for many Black youths in the US,^12,13^ and school segregation has increased in recent decades. Understanding the processes by which school segregation contributes to differences in cognition (eg, school quality; adolescent stress) is important for addressing disparities in late-life cognition for the next generation.

## Notes

Supplement. **eTable 1.** States Included in the Southern Birth Region, Based on US Census Classifications **eTable 2.** Demographic Comparison of Participants Included in the Primary Analyses, and Those Excluded for Missing Information on School Desegregation at 3 or More Time Points **eTable 3.** Linear Regression Coefficients for the Association Between Segregated/Integrated School Attendance Experience or Missing School Information and *z* Standardized Late-Life Cognitive Domains in STAR **eTable 4.** Linear Regression Coefficients for the Association Between Segregated/Integrated School Attendance Experience and *z* Standardized Late-Life Cognitive Domains in STAR **eTable 5.** Linear Regression Coefficients for the Association Between Segregated/Integrated School Attendance Experience and *z* Standardized Late-Life Cognitive Domains in STAR
