# Supplementary material for: Association of Timing of School Desegregation in the United States With Late-Life Cognition in the Study of Healthy Aging in African Americans (STAR) Cohort
Source: JAMA Netw Open. 2021 Oct 20;4(10):e2129052. doi: 10.1001/jamanetworkopen.2021.29052 (PMC8529411; doi:10.1001/jamanetworkopen.2021.29052)
Supplement: Supplement. — eTable 1. States Included in the Southern Birth Region, Based on US Census Classifications eTable 2. Demographic Comparison of Participants Included in the Primary Analyses, and Those Excluded for Missing Information on School Desegregation at 3 or More Time Points eTable 3. Linear Regression Coefficients for the Association Between Segregated/Integrated School Attendance Experience or Missing School Information and z Standardized Late-Life Cognitive Domains in STAR eTable 4. Linear Regression Coefficients for the Association Between Segregated/Integrated School Attendance Experience and z Standardized Late-Life Cognitive Domains in STAR eTable 5. Linear Regression Coefficients for the Association Between Segregated/Integrated School Attendance Experience and z Standardized Late-Life Cognitive Domains in STAR [file jamanetwopen-e2129052-s001.pdf]

# Supplementary Online Content

Peterson RL, George KM, Barnes LL, et al. Association of timing of school desegregation in the United States with late-life cognition in the Study of Healthy Aging in African Americans (STAR) cohort. *JAMA Netw Open*. 2021;4(10):e2129052. doi:10.1001/jamanetworkopen.2021.29052

**eTable 1.** States Included in the Southern Birth Region, Based on US Census Classifications
**eTable 2.** Demographic Comparison of Participants Included in the Primary Analyses, and Those Excluded for Missing Information on School Desegregation at 3 or More Time Points
**eTable 3.** Linear Regression Coefficients for the Association Between Segregated/Integrated School Attendance Experience or Missing School Information and *z* Standardized Late-Life Cognitive Domains in STAR
**eTable 4.** Linear Regression Coefficients for the Association Between Segregated/Integrated School Attendance Experience and *z* Standardized Late-Life Cognitive Domains in STAR
**eTable 5.** Linear Regression Coefficients for the Association Between Segregated/Integrated School Attendance Experience and *z* Standardized Late-Life Cognitive Domains in STAR

This supplementary material has been provided by the authors to give readers additional information about their work.

© 2021 Peterson RL et al. *JAMA Network Open*.

**eTable 1.** States Included in the Southern Birth Region, Based on US Census Classifications

| | | |
|---|---|---|
| Alabama | Kentucky | South Carolina |
| Arkansas | Louisiana | Tennessee |
| Delaware | Maryland | Texas |
| District of Columbia | Mississippi | Virginia |
| Florida | North Carolina | West Virginia |
| Georgia | Oklahoma | |

© 2021 Peterson RL et al. *JAMA Network Open*.

**eTable 2.** Demographic Comparison of Participants Included in the Primary Analyses, and Those Excluded for Missing Information on School Desegregation at 3 or More Time Points

| | **Primary Analytic Sample (699)** | **Missing School Info (44)** | **p-value test of difference** |
|---|---|---|---|
| Age | 68.5 (8.6) | 72.5 (9.8) | 0.002* |
| Female | 484 (69.2) | 31 (70.5) | 0.87ŧ |
| **Highest Education, mean (SD)** | 14.5 (2.4) | 12.9 (2.8) | <0.001* |
| Grade school | 0 | 3 (6.12%) | <0.001 ŧ |
| Some High School | 6 (0.9%) | 10 (20.4%) | |
| High School/GED | 102 (14.6%) | 11 (22.5%) | |
| Tech/Trade School | 27 (3.9%) | 4 (8.2%) | |
| Some College | 310 (44.4%) | 11 (22.5%) | |
| College | 124 (17.7%) | 6 (12.2%) | |
| Graduate School | 130 (18.6%) | 4 (8.2%) | |
| Executive Function | 0.04 (.97) | -0.35 (1.3) | 0.01* |
| Verbal Episodic Memory | 0.04 (0.98) | -0.45 (1.12) | <0.001* |
| Semantic Memory | 0.05 (0.97) | -0.58 (1.16) | <0.001* |
| Southern Birth State | 257 (36.8%) | 22 (50%) | 0.08ŧ |

*One-way ANOVA; ŧ $\chi^2$ test of significance

© 2021 Peterson RL et al. *JAMA Network Open*.

**eTable 3.** Linear Regression Coefficients for the Association Between Segregated/Integrated School Attendance Experience or Missing School Information and *z* Standardized Late-Life Cognitive Domains in STAR. Models control for age, gender, participant education and birth region.

| | **Executive Function** | **Semantic Memory** | **Verbal Episodic Memory** |
|---|---|---|---|
| | **β ( 95% CI)** | **β ( 95% CI)** | **β ( 95% CI)** |
| Never attended integrated schools | ref. | ref. | ref. |
| Only attended integrated schools | 0.13 (-0.06 - 0.32) | 0.34 (0.14 - 0.54) | 0.00 (-0.20 - 0.21) |
| Integrated between 1st and 6th grades | 0.34 (0.08 - 0.61) | 0.43 (0.15 - 0.72) | 0.07 (-0.22 - 0.35) |
| Integrated between 6th and 9th grades | -0.1 (-0.37 - 0.17) | -0.01 (-0.30 - 0.28) | -0.04 (-0.33 - 0.25) |
| Integrated between 9th and 12th grades | 0.06 (-0.26 - 0.39) | 0.21 (-0.14 - 0.56) | 0.27 (-0.07 - 0.62) |
| From integrated to segregated schools | 0.06 (-0.29 - 0.40) | 0.54 (0.16 - 0.91) | 0.08 (-0.29 - 0.45) |
| Missing school segregation information | 0.19 (-0.09 - 0.47) | 0.00 (-0.30 - 0.30) | -0.14 (-0.45 - 0.16) |

© 2021 Peterson RL et al. *JAMA Network Open*.

**eTable 4.** Linear Regression Coefficients for the Association Between Segregated/Integrated School Attendance Experience and *z* Standardized Late-Life Cognitive Domains in STAR. Models control for age, gender, participant education, birth region, childhood hunger, childhood family finances and parental education.

| | **Executive Function** | **Semantic Memory** | **Verbal Episodic Memory** |
|---|---|---|---|
| | **β (95% CI)** | **β (95% CI)** | **β (95% CI)** |
| Never attended integrated schools | ref. | ref. | ref. |
| Only attended integrated schools | 0.09 (-0.12 - 0.29) | 0.18 (-0.03 - 0.40) | -0.01 (-0.23 - 0.21) |
| Integrated between 1st and 6th grades | 0.34 (0.05 - 0.62) | 0.36 (0.06 - 0.66) | 0.04 (-0.27 - 0.35) |
| Integrated between 6th and 9th grades | -0.07 (-0.37 - 0.22) | -0.09 (-0.40 - 0.22) | 0.01 (-0.31 - 0.33) |
| Integrated between 9th and 12th grades | -0.15 (-0.49 - 0.19) | 0.01 (-0.35 - 0.36) | 0.16 (-0.20 - 0.53) |
| From integrated to segregated schools | 0.08 (-0.29 - 0.45) | 0.45 (0.06 - 0.85) | 0.06 (-0.34 - 0.47) |

© 2021 Peterson RL et al. *JAMA Network Open*.

**eTable 5.** Linear Regression Coefficients for the Association Between Segregated/Integrated School Attendance Experience and *z* Standardized Late-Life Cognitive Domains in STAR. Models control for age, gender, and birth region.

| | **Executive Function** | **Semantic Memory** | **Verbal Episodic Memory** |
|---|---|---|---|
| | **β (95% CI)** | **β (95% CI)** | **β (95% CI)** |
| Never attended integrated schools | ref. | ref. | ref. |
| Only attended integrated schools | 0.13 (-0.07 - 0.34) | 0.33 (0.12 - 0.54) | -0.01 (-0.22 - 0.20) |
| Integrated between 1st and 6th grades | 0.30 (0.02 - 0.58) | 0.40 (0.10 - 0.69) | 0.04 (-0.25 - 0.34) |
| Integrated between 6th and 9th grades | -0.19 (-0.48 - 0.10) | -0.08 (-0.38 - 0.21) | -0.09 (-0.38 - 0.21) |
| Integrated between 9th and 12th grades | 0.04 (-0.30 - 0.39) | 0.19 (-0.16 - 0.55) | 0.26 (-0.09 - 0.62) |
| From integrated to segregated schools | 0.07 (-0.30 - 0.44) | 0.53 (0.14 - 0.92) | 0.08 (-0.30 - 0.46) |

© 2021 Peterson RL et al. *JAMA Network Open*.
